# Examining Changes in Shoulder Strength, Lower Body Power, and Body Composition among Collegiate Baseball Players after Completion of a Summer Baseball League Season

**DOI:** 10.3390/jfmk9020098

**Published:** 2024-05-31

**Authors:** Brandon Merfeld, Matthew Rowley, Thomas Almonroeder, Joel Luedke, Jacob L. Erickson, Margaret T. Jones, Jennifer B. Fields, Elijah Szymanski, Andrew R. Jagim

**Affiliations:** 1 Biology Department, St. Mary’s University, Winona, MN 55987, USA; brmerf18@smumn.edu (B.M.);; 2 Department of Physical Therapy, Trine University, Angola, IN 46703, USA; 3 Sports Medicine, Mayo Clinic Health System, La Crosse, WI 54601, USA; 4 Patriot Performance Laboratory, Frank Pettrone Center for Sports Performance, George Mason University, Fairfax, VA 22030, USA; 5 Sport, Recreation, and Tourism Management, George Mason University, Fairfax, VA 22030, USA; 6 Department of Nutritional Sciences, University of Connecticut, Storrs, CT 06269, USA; 7 Medical College of Wisconsin-Central Wisconsin, Wausau, WI 54401, USA

**Keywords:** arm strength, throwing arm, athlete monitoring, overhead athletes

## Abstract

The strength of the shoulder musculature involved with internal rotation and arm extension plays an important role in the overhead throwing motion for baseball athletes, both for throwing-related performance and injury risk. The maintenance of shoulder strength is a high priority for baseball athletes throughout a season; however, little is known in regards to the expected changes in strength throughout a season. To examine pre-post changes in shoulder strength, lower body power, and body composition among collegiate baseball players after the completion of a summer baseball league season. Amateur baseball players (*n* = 12; age: 20.9 ± 1.0 years.; height: 181.6 ± 5.6 cm; body mass: 86.4 ± 11.1 kg; BMI: 26.0 ± 2.6 kg/m^2^) participated in the current study. Pre- and post-competitive season, the participants completed shoulder strength assessments and body composition and countermovement vertical jump (CMJ) tests. An upper-body isometric test (athletic shoulder [ASH] test) was used to evaluate shoulder strength for each arm. Each subject completed maximal isometric contractions for both the throwing and non-throwing arms at four separate angles of abduction (180°, ‘I’; 135°, ‘Y’; 90°, ‘T’; and −180°, ‘A’) while lying in a prone position. For shoulder strength, the primary dependent variable of interest was a composite measure that represented the average of the forces produced across all four positions of the ASH test (I, Y, T, A). For the ASH test composite measure, there was a trend toward a significant arm-by-time interaction effect (*p* = 0.08), as shoulder strength decreased by 9.03% for the throwing arm (ES = 0.72; 95% CI = [−0.27, −0.01]), compared to only 2.03% for the non-throwing arm (ES = 0.15; 95% CI = [−0.16, 0.09]), over the course of the season. The main effects of time (*p* = 0.16) and arm (*p* = 0.58) were not significant for the ASH test composite measure. There was no relationship between lower body power and throwing arm strength at baseline (r = 0.20, *p* = 0.56), and only a non-significant weak relationship at post-test (r = 0.28, *p* = 0.41). Throughout a season, baseball players may experience reductions in shoulder strength of the throwing arm with minimal changes in shoulder strength in the non-throwing arm.

## 1. Introduction

The strength of the shoulder musculature involved with internal rotation and arm extension is positively associated with baseball throwing velocity [1,2]. Shoulder strength, stability, and range of motion have all been shown to influence throwing biomechanics, in which certain deficiencies may predispose baseball players to arm injuries [3,4,5]. The prevalence of such arm injuries has grown exponentially across multiple age groups, especially injuries of the ulnar collateral ligament [4,6]. This increase is thought to be attributable to an emphasis on throwing velocity, as well as an increase in the number of games played, innings pitched, higher pitch counts, and early sport specialization [4,6,7,8]. Previous research has demonstrated an arm injury rate of 2.22/1000, 2.50/1000, and 1.81/1000 per athletic exposure for youth, high school, and collegiate baseball players, respectively [9]. Research efforts have explored the identification of risk factors for baseball-related arm injuries [4] and the efficacy of preventative measures [10] to attenuate the risks associated with them. In addition to the mitigation of injury risk, baseball players, coaches, and performance staff seek opportunities to optimize and monitor changes in arm strength to facilitate performance improvements, namely increased throwing velocity [11].

Performance and functional assessments can serve to evaluate baseball players’ potential for on-field success and help with the identification of risk factors for injuries. Pre-season measures of shoulder strength levels (prone external rotation, seated external rotation, and supraspinatus strength) have been found to be associated with throwing-related injuries requiring surgical interventions [12]. Moreover, the internal rotational torque during the arm cocking phase and the compressive forces occurring during the deceleration phase in throwing are thought to play a causative role in arm pathology [3,13]. As such, strengthening the external rotator and internal rotator musculature of the shoulder has been identified as a promising injury-risk reduction strategy [2,10,11]. Recently, the athletic shoulder test was found to demonstrate an excellent interday reliability net peak force (ICC 0.94–0.98) and high absolute reliability values (standard error of measurement: 4.8–10.8), lending itself to serve as a valid tool for isometrically evaluating shoulder strength in athletes [14], particularly in baseball players [15]. This assessment offers a non-invasive field-based technique to evaluate the changes in shoulder strength for throwing performance and helps to identify potential risk factors for injuries, without the need for costly laboratory equipment. Additionally, there are known differences in shoulder strength across different arm positions [15,16], between pitchers and position players [17], and between throwing and non-throwing arms [15,18] across different levels of competition. An established understanding of the relationships between measures of shoulder strength and throwing biomechanics can provide targeted strategies for arm-focused strength training programs and load-monitoring strategies to improve throwing velocity and mitigate the risk of injury.

The movement kinetics and kinematics of the overhead throwing motion indicate that upper and lower extremity strength is important for throwing velocity and injury risk reduction [19]. From a performance standpoint, several studies have identified relationships between higher pitching velocities and lower body power at youth [20,21], high school [22], collegiate [19,23], and minor league levels [24]. Previous research has indicated that the lower body is primarily responsible for the initiation of trunk rotation, generating power at the beginning of the throwing motion, and for absorbing much of the excess energy nearing the end of a pitch [20,25,26,27]. The lower body facilitates the energy transfer of rotational forces up the kinetic chain [26]. This relationship highlights the importance of developing lower body power to maximize an athlete’s throwing ability and reduce the stress placed on the arm when throwing. 

With the high prevalence of arm injuries in baseball, it is important to examine the longitudinal changes in arm strength that may occur throughout a season. Moreover, a better understanding of the differences in shoulder strength across position groups and between the throwing arm versus non-throwing arm can help direct individualized strength and conditioning programming. Currently, a paucity of literature exists regarding measures of shoulder strength in collegiate baseball players, yet more than 25,000 baseball players are competing at the collegiate level in the United States [28]. Therefore, the primary purpose of this study was to examine pre-post changes in shoulder strength, lower body power, and body composition among collegiate baseball players after the completion of a summer baseball league season. In addition, the relationship between shoulder strength, body composition, and lower body power was also examined.

## 2. Methods

### 2.1. Subjects

Collegiate male baseball players (*n* = 12) were recruited to participate in the current study. Only players who participated in more than 75% of innings throughotut the summer season were included in the final analysis. Players were categorized as pitchers (*n* = 6) or non-pitchers (*n* = 6). The inclusion criteria included medical clearance to participate in baseball activities, being between the ages of 18–26 years, and classified as a starter on the team. The exclusion criteria included a diagnosis with a current injury that prevented participation in summer baseball activities. All players had recently completed their collegiate baseball season prior to participating in the summer league. The study was approved and conducted under the guidelines of the Institutional Review Board and written informed consent was obtained from all subjects prior to data collection. 

### 2.2. Study Design

For this observational study, collegiate-level baseball players competing in an amateur summer baseball league completed testing sessions before (baseline) and after (post) a 12-week, 32-game competitive season with 24 practices. The players were not participating in any other organized baseball or strength and conditioning activities throughout the summer season. All baseline testing occurred within 1 week of the start of the season and post-testing was completed within 1 week of the last game. The testing sessions consisted of assessments of shoulder strength for the throwing and non-throwing arms, lower body power, and body composition. The primary outcome measures included changes in peak force for each arm position during the ASH test. Secondary outcome measures included changes in lower body power and body composition. A single member of the research team conducted all participant evaluations.

### 2.3. Data Collection Procedures

#### 2.3.1. Anthropometrics and Body Composition

Prior to testing, the athletes were instructed to refrain from exercise, eating, and drinking for >2 h before the scheduled study visit. Body mass and height were assessed using a self-calibrating physician’s scale and stadiometer to the nearest 0.1 kg and 0.5 cm, respectively. Body composition was assessed by bioimpedance spectroscopy (BIS) using the SFB7 (ImpediMed Limited, Pinkenba, QLD, Australia) device for the determination of fat mass, fat-free mass (FFM), and body fat percentage. Previous research has found BIS to provide a valid measure of FFM in young-adult male athletes (r = 0.62; SEM = 1.03 kg) [29]. For this test, the subjects remained in a supine position for 5 min before each measurement. Before each analysis, the subject’s age, height, and weight were entered into the device. The subjects were then asked to remain in the supine position, with arms at approximately 30° from the torso. Two electrodes were then placed on their right hand and foot according to manufacturer recommendations.

#### 2.3.2. Shoulder Strength

**Figure 1:**
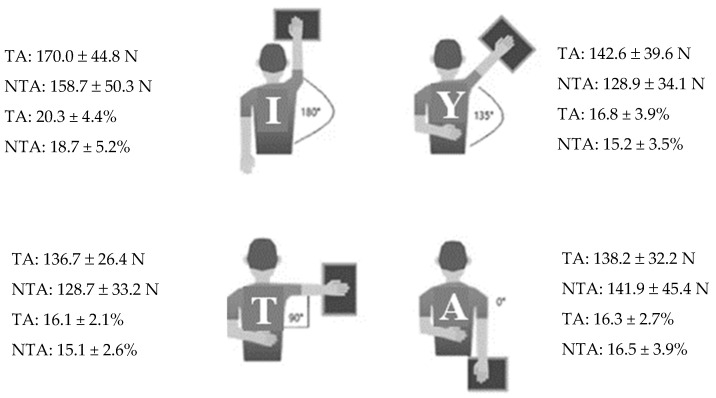
Subject completing the I (top-left), Y (top-right), T (bottom-left), A (bottom-right) components of the athletic shoulder (ASH) test. TA = throwing arm; NTA = non-throwing arm; % = percentage of body mass.

An upper-body isometric test (athletic shoulder [ASH] test) was used to evaluate shoulder strength for each limb in accordance with Ashworth et al. [14]. Each subject completed maximal isometric contractions for both the throwing and non-throwing arms at four separate angles of abduction (180°, ‘I’; 135°, ‘Y’; 90°, ‘T’; and 0°, ‘A’) while lying in a prone position, with a pillow resting under the forehead (Figure 1). Three trials were conducted per arm, in each position, with the strength values averaged across the three trials. During each trial, the subject was instructed to push as hard as possible for three seconds against a force plate that sampled at 1000 Hz (Hawkins Dynamics, Westbrook, ME, USA). The contralateral arm was placed behind the back so that the arm was unable to provide anti-rotation trunk stability for the Y, T, and A positions. For the I position, the contralateral arm was allowed to remain by the subject’s side due to comfort and proper position. Upon completion of the test, the data were automatically exported to a cloud-based platform for later analysis. For shoulder strength, the primary dependent variable of interest was a composite measure that represented the average of the forces produced across all four positions of the ASH test (I, Y, T, A) [14]. This ASH test composite measure represents overall shoulder strength across the different testing positions. The ASH test measures for the throwing and non-throwing arms, and a ratio that reflected the strength for the throwing arm relative to the non-throwing arm was also examined. The strength measures for each position of the ASH test (I, Y, T, A) were also evaluated and the ASH test forces were normalized by body mass (N/kg).

#### 2.3.3. Lower Body Power

Lower body power was assessed based on countermovement vertical jumps on bilateral force platforms, with sample ground reaction force data at 1000 Hz (Hawkins Dynamics, USA) [30,31]. For each trial, the subjects were instructed to perform a rapid countermovement to a self-selected depth and then jump vertically as high as possible. The subjects were instructed to start with their hands on their hips but were then allowed to swing their arms in counter-movement fashion as they performed the jump. The subjects were also instructed to stick and hold the landing. The subjects completed three maximal effort attempts, with 2 min of rest in between attempts.

The vertical ground reaction force data recorded during the countermovement jumps was used to calculate the velocity of the center of mass, by first finding the center of mass acceleration by dividing the vertical ground reaction force (minus bodyweight) by body mass and then numerically integrating the acceleration with respect to time using the trapezoidal method [30,31]. The subject’s bodyweight was obtained as they stood motionless on the force plates prior to initiating each trial. Lower body power was calculated by finding the product of the vertical ground reaction force and the center of mass velocity at each time point during the countermovement jump. Peak power was identified for each countermovement jump and averaged across the three trials. The manufacturer’s proprietary software program was used to process the ground reaction force data.

### 2.4. Statistical Analyses

For the ASH test composite variable, a mixed-model ANOVA was conducted, with a between-subjects factor of “group” (pitchers, non-pitchers) and within-subjects factors of “time” (baseline, post) and “arm” (throwing, non-throwing). The factor “group” did not exhibit any significant interaction or main effects; therefore, the “group” factor was removed from the statistical model and collapsed data across position groups in order to simplify interpretation of results. The mixed-model ANOVA with the factors group and time were conducted for the ASH test composite measure ratios (strength of the throwing arm relative to the non-throwing arm), body composition variables, and lower body power. An alpha level of 0.05 was used for the determination of statistical significance. Cohen’s d effect sizes (ES) and 95% confidence intervals (95% CI) were generated for each pairwise comparison. The effect sizes were calculated by dividing the mean difference (post vs. baseline) by the average of the standard deviations from the baseline and post time points. The effect sizes were interpreted using the following criteria: 0.2 = trivial, 0.2–0.6 = small, 0.7–1.2 = moderate, 1.3–2.0 = large, and >2.0 = very large [28]. The percent change was calculated based on the baseline and post-testing means. Spearman’s correlation was used to examine the relationship between lower body power and shoulder strength for the throwing arm (based on the ASH test composite measure). The following criteria were used to evaluate the correlation coefficients (r values): 0–0.25 = little or no relationship, 0.25–0.50 = weak relationship, 0.50–0.75 = moderate relationship, >0.75 = strong relationship [32]. All data were analyzed using jamovi software (https://www.jamovi.org/ (accessed on 18 April 2024)).

## 3. Results

Twelve NCAA Division II and III men’s collegiate baseball players competing in a summer amateur league (*n* = 12; age: 20.9 ± 1.0 years.; height: 181.6 ± 5.6 cm; body mass: 86.4 ± 11.1 kg; BMI: 26.0 ± 2.6 kg/m^2^) participated in the current study.

### 3.1. Shoulder Strength

At baseline, no significant differences (*p* > 0.05) in peak force were observed between the throwing arm and non-throwing arm for any of the arm positions (Figure 1 and Table 1).

**Figure 2:**
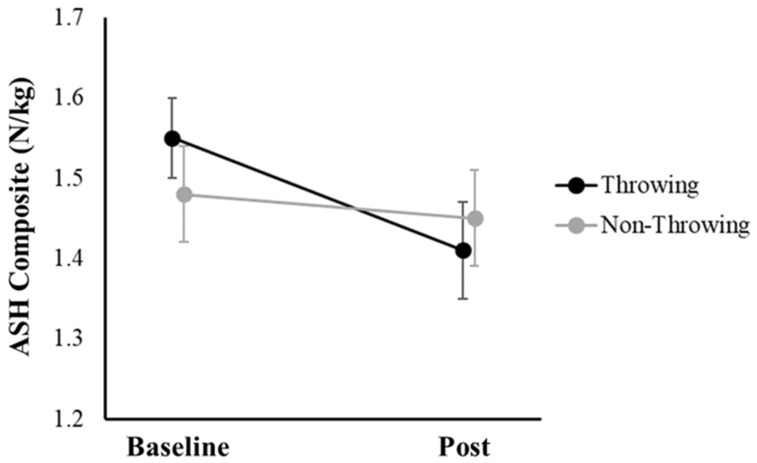
Means (±standard errors) for the athletic shoulder (ASH) test composite measure for the throwing (black) and non-throwing arms (grey), before (baseline) and immediately after (post) the season.

For the ASH test composite measure, there was a trend toward a significant arm-by-time interaction effect (*p* = 0.08), as shoulder strength decreased by 9.03% for the throwing arm (ES = 0.72; 95% CI = [−0.27, −0.01]) compared to 2.03% for the non-throwing arm (ES = 0.15; 95% CI = [−0.16, 0.09]), over the course of the season (Figure 2; Table 2). The main effects of time (*p* = 0.16) and arm (*p* = 0.58) were not significant for the ASH test composite measure. 

For the throwing arm, the reductions in shoulder strength ranged from 6.80% for the Y position to 12.75% for the A position (Table 2). For the non-throwing arm, shoulder strength remained unchanged over time for the Y position (0% change), increased by 2.90% for the T position, and decreased by 3.01% for the I position and 8.05% for the A position (Table 2). 

For the ASH test composite measure ratios, there was not a significant group-by-time interaction effect (*p* = 0.16) or a main effect of group (*p* = 0.18). There was a trend toward a significant main effect of time (*p* = 0.06) as the shoulder strength ratios tended to decrease (1.05 ± 0.08 vs. 0.97 ± 0.08), reflecting a reduction in strength for the throwing arm relative to the non-throwing arm. However, the main effect of time was not statistically significant (*p* = 0.06; ES = 1.00; 95% CI = [−0.15, −0.01]).

### 3.2. Body Composition

There was not a significant group-by-time interaction effect (*p* ≥ 0.41) or main effects of group (*p* ≥ 0.13) or time (*p* ≥ 0.87) for the body composition variables (body fat percentage, fat-free mass) (Table 3).

### 3.3. Lower Body Power

For lower body power, there was not a significant group-by-time interaction effect (*p* = 0.30) or main effect of group (*p* = 0.48) (Table 3); however, there was a main effect of time (*p* = 0.01), as the lower body increased by 44.79% from baseline to post-testing (ES = 1.41; 95% CI = [7.36, 26.36]).

### 3.4. Relationship between Lower Body Power and Throwing Arm Strength

There was no relationship between lower body power and throwing arm strength at the baseline (r = 0.20, *p* = 0.56), and only a non-significant weak relationship at post-test (r = 0.28, *p* = 0.41). There also was no relationship between the change in lower body power and the change in throwing arm strength (r = −0.06, *p* = 0.85).

## 4. Discussion

The primary aim of the current study was to evaluate changes in shoulder strength over the course of the summer baseball league season. The main findings indicate that strength changes are arm dependent. Specifically, decreases in strength were significantly greater in the throwing arm compared to the non-throwing arm, which suggests that a season of throwing may result in arm fatigue, thereby contributing to reductions in shoulder strength. Additionally, there was no relationship between the change in lower body power and the change in throwing arm strength.

Shoulder strength has shown to be a predictor of injury risk in baseball players [12] and is often a target for sport-specific baseball training and strength and conditioning activities as it has also been associated with faster throwing velocities [1,33]. In the current study, there were significant reductions in throwing arm shoulder strength, which ranged from 6.80% for the Y position to 12.75% for the A position (Table 2). For the non-throwing arm, shoulder strength remained unchanged over time for the Y position (0% change), increased by 2.90% for the T position, and decreased by 3.01% for the I position and 8.05% for the A position. For the ASH test composite measure ratios, there was no group-by-time interaction effect or main effect of group. There was a trend toward a significant main effect of time, as the shoulder strength ratios tended to decrease over time (1.05 ± 0.08 vs. 0.97 ± 0.08), reflecting a reduction in strength for the throwing arm relative to the non-throwing arm. These findings are in support of previous work by Downs et al. [34], who proposed that shoulder fatigue, likely a result of high-volume throwing and concomitant training activities throughout a season, subsequently contributed to reductions in shoulder strength. Specifically, Downs et al. [34] observed reductions in bilateral shoulder internal rotation isometric strength following a season. Interestingly, a pre-throwing arm-strengthening program was employed by the intervention group throughout the season; however, the intervention group still experienced a greater reduction in strength compared to the control group. Despite the shorter duration and game frequency of the baseball summer league season in the current study compared to a traditional collegiate season, there were still notable reductions in shoulder strength in the throwing arm, yet minimal reductions in shoulder strength were observed in the non-throwing arm. Future research examining the efficacy of arm-strengthening activities (e.g., resistance band warm-up routines) and load management strategies (e.g., pitch counts, bullpen sessions, long toss) for the maintenance and development of shoulder strength is warranted.

Findings from the current study indicate that there were no differences in shoulder strength for any of the arm positions when comparing the throwing arm to the non-throwing. In contrast, several studies have reported differences in shoulder strength between the throwing and non-throwing arm, with the throwing arm often displaying greater indices of strength [35]. However, these differences may be influenced by the specific strength measure, assessment modality, time of year, and population being evaluated [36,37,38]. For example, Hurd et al. [37] and Mulligan et al. [38] observed less shoulder external rotation strength for the throwing arm compared to the non-throwing arm in high school baseball athletes; however, the opposite was true for internal rotation strength. Donatelli et al. [35] reported significantly stronger middle trapezius muscles (Pitching: 6.66 ± 1.7 kg, vs. Non-pitching: 5.84 ± 1.7 kg), lower trapezius muscles (Pitching: 6.85 ± 1.9 kg, vs. Non-pitching: 6.08 ± 1.2 kg), and internal rotators in 90° of abduction (Pitching: 18.2 ± 3.9 kg, vs. Non-pitching: 17.43 ± 3.7 kg) in the pitching arm compared to non-pitching as determined by a handheld dynamometer in professional baseball pitchers. Additionally, the authors did find the pitching arm’s external rotators to be significantly weaker than those of the nonpitching arm, when tested in the plane of scapula (Pitching: 13.3 ± 3.6 kg, vs. Non-pitching: 14.5 ± 3.1 kg) and in 90° of abduction (Pitching: 15.1 ± 3.7 kg, vs. Non-pitching: 17.1 ± 4.1 kg). Trakis et al. [36] reported arm pain to be an important indicator of strength imbalances in adolescent pitchers between the pitching and non-pitching arm. Specifically, the authors reported muscle strength in the pitching-arm versus non-pitching arm to be lower in athletes who reported arm pain versus the non-pain group for the middle trapezius (7% ± 19% vs. 22% ± 12%; *p* < 0.05) and supraspinatus (−4% ± 27% vs. 14% ± 14%; *p* < 0.05). Alternatively, the non-pitching arm was stronger for the internal rotators (19% ± 14% vs. 6% ± 12%; *p* < 0.05).

The majority of the studies have utilized isokinetic strength tests or internal/external rotational strength of the shoulder musculature to evaluate shoulder strength [35,38], whereas the current study employed force plates to evaluate isometric shoulder strength. It is possible that shoulder strength differences may not be as robust for isometric strength evaluations as compared to movement-based dynamic strength evaluations used during isokinetic strength assessments. Small effect sizes were observed, indicating that minimal differences were detected for measures of shoulder strength between the throwing and the non-throwing arm at each arm position (Table 2). Additionally, the ratio between the throwing arm and non-throwing arm was >1.0 for each arm position, providing further evidence that the throwing arm has a greater magnitude of isometric shoulder strength at each arm position. Moreover, while minimal differences in shoulder strength were observed in the current study, specific strength measures of the internal rotator and external rotator musculature were not evaluated. Conversely, previous research has noted differences in external rotator strength for the throwing arm compared to the non-throwing arm, often indicating a muscle imbalance of antagonistic muscle groups of the shoulder musculature [39,40,41]. It is thought the repetitive throwing motion results in muscular weakness and atrophy of the infraspinatus, potentially mediated by nerve impingement and infrequent external rotation motion [41,42]. Interestingly, the shoulder strength values derived from the ASH test in the current study are higher than those previously reported in NCAA Division II baseball players, for both throwing and non-throwing arm and across all arm positions [15].

A secondary aim of the current study was to evaluate the potential relationships between shoulder strength, body composition, and lower body power. It is well known that lower body strength and greater ground reaction forces produced during the throwing motion are consistently related to throwing velocity in baseball players [22,23,43,44]. However, less is known regarding the relationship between lower body power and shoulder strength. Despite previous research indicating strong associations between lower body power, throwing kinematics, and pitch velocity [19,20,21,22,23] along with measures of batting performances in baseball [45], findings from the current study did not indicate a relationship between lower body power and throwing arm strength (based on the ASH test composite measure) at baseline and only a non-significant weak relationship at post-testing. It is possible the previously noted relationships between lower body power and baseball-specific performances exist independently of isolated measures of shoulder strength. It is unknown why such large improvements in lower body power were observed throughout the season. It is possible the players were experiencing fatigue from their collegiate season and were able to recover and regain lower body power as the summer season went on. Additionally, the observed reductions in arm strength following the season from the current study are likely not a result of reductions in lower body power or body composition, as there were no relationships between the changes in lower body power and the changes in throwing arm strength or body composition. It is unknown if similar changes in arm strength may occur at different levels of competition such as at high school or professional level or in other throwing sports such as softball.

This study is not without limitations. The small sample size may have limited the ability to detect differences in shoulder strength. Additionally, the lack of isolated external rotator and internal rotator strength measures precludes the ability to examine musculature imbalances within the same arm. Lastly, the lack of information regarding pitch counts, throwing velocity, and throwing workloads is another limitation of the study as these measures of workload may have implications for shoulder strength throughout a baseball season.

## 5. Conclusions

Reductions in throwing arm strength occur throughout a season in collegiate baseball players. Therefore, a primary goal of the offseason should be to build shoulder strength and stability when less stress is placed on the shoulder joint due to the lower volume of throwing. An emphasis should be placed on training both the external and internal rotator musculature of the shoulder as this has been identified as an injury-prevention strategy for baseball players.

While the priority is to build strength and stability in the shoulder during the offseason, the primary focus throughout the season should be to maintain strength in the shoulder and manage pitch counts (pitchers only) or throwing load on the arm for positional players. To reduce injury risk, athletes should be closely monitored when attempting to increase throwing velocity. Practitioners can work to increase lower body power to maximize an athlete’s throwing ability and reduce the stress placed on the arm during throwing activities. Further, an arm-focused strength training program and load-monitoring practices are essential to improve throwing velocity and mitigate the risk of injury.

## Figures and Tables

**Table 1:** Descriptive summary of peak force values for each arm position.

	Throwing Arm	Non-Throwing Arm	Difference	%	Ratio	*p* Value	ES (95% CI)
I Position (N)	170.0 ± 44.8	158.7 ± 50.3	13.3 ± 32.9	12.5 ± 27.9	1.12 ± 0.28	0.155	0.40 (−0.15, 0.94)
Y Position (N)	142.6 ± 39.6	128.9 ± 34.1	13.6 ± 25.8	12.1 ± 21.0	1.12 ± 0.21	0.069	0.53 (−0.40, 1.08)
T Position (N)	136.7 ± 26.4	128.7 ± 33.2	8.0 ± 18.2	8.6 ± 16.7	1.09 ± 0.17	0.124	0.44 (−0.12, 0.98)
A Position (N)	138.2 ± 32.2	141.9 ± 45.4	−3.7 ± 28.7	1.5 ± 18.7	1.09 ± 0.18	0.636	0.13 (−0.65, 0.39)

Data are mean ± SD. N = newtons; Athletic shoulder test positions: 180°, ‘I’; 135°, ‘Y’; 90°, ‘T’ and 0°, ‘A’.

**Table 2:** Shoulder strength descriptive statistics.

	Baseline	Post	ES	% Change	95% CI
Composite—Throwing	1.55 ± 0.18	1.41 ± 0.21	0.72	−9.03	−0.27, −0.01 *
Composite—Non-Throwing	1.48 ± 0.21	1.45 ± 0.20	0.15	−2.03	−0.16, 0.09
I Position—Throwing	1.73 ± 0.29	1.61 ± 0.35	0.38	−6.94	−0.37, 0.13
I Position—Non-Throwing	1.66 ± 0.31	1.61 ± 0.26	0.18	−3.01	−0.16, 0.06
Y Position—Throwing	1.47 ± 0.28	1.37 ± 0.28	0.36	−6.80	−0.33, 0.13
Y Position—Non-Throwing	1.39 ± 0.27	1.39 ± 0.17	0.00	0.00	−0.16, 0.17
T Position—Throwing	1.50 ± 0.15	1.34 ± 0.17	1.00	−10.67	−0.26, −0.05 *
T Position—Non-Throwing	1.38 ± 0.20	1.42 ± 0.25	0.18	2.90	−0.13, 0.20
A Position—Throwing	1.49 ± 0.23	1.30 ± 0.22	0.84	−12.75	−0.32, −0.06 *
A Position—Non-Throwing	1.49 ± 0.31	1.37 ± 0.30	0.39	−8.05	−0.30, 0.06

Mean ± standard deviation ASH test shoulder strength measures (N/kg); ES = Cohen’s d effect sizes; % Change = percent change (post—baseline); Negative values reflect decreased shoulder strength, positive values reflect increased shoulder strength; 95% CI = 95% confidence interval based on changes over time (post—baseline); * Indicates confidence interval does not include 0.

**Table 3:** Body composition and lower body power descriptive statistics.

	Baseline	Post	ES	% Change	95% CI
Body fat (%)	11.36 ± 7.20	11.04 ± 5.57	0.05	−2.82	−4.05, 3.42
Fat-free mass (kg)	76.08 ± 9.56	76.04 ± 12.44	0.00	−0.05	−4.40, 4.33
Lower body power (W/kg)	37.64 ± 9.24	54.50 ± 14.71	1.41	44.79	7.36, 26.36

Mean ± standard deviation; ES = Cohen’s d effect sizes; % Change = percent change (post—baseline); 95% CI = 95% confidence interval based on changes over time (post—baseline).

## Data Availability

The data presented in this study are available on request from the corresponding author.
